# The relationship between attention deficit hyperactivity disorder and premature infants in Taiwanese: a case control study

**DOI:** 10.1186/1471-244X-12-85

**Published:** 2012-07-23

**Authors:** Shih-Ming Chu, Ming-Horng Tsai, Fan-Ming Hwang, Jen-Fu Hsu, Hsuan Rong Huang, Yu-Shu Huang

**Affiliations:** 1 Division of Pediatric Neonatology, Department of Pediatrics, Chang Gung Memorial Hospital, Taoyuan, Taiwan; 2 Department of Education, National Chia-Yi University, Chiayi, Taiwan; 3 Division of Pediatric Neonatology and Hematology/Oncology, Chang Gung Memorial Hospital, Yunlin, Taiwan; 4 Department of Child Psychiatry and Sleep Center, Chang Gung Memorial Hospital, Taoyuan, Taiwan; 5 College of Medicine, Chang Gung University, Taoyuan, Taiwan; 6 Chang Gung Institute of Technology, Chiayi, Taiwan; 7 Department of Child Psychiatry and Sleep Center, Chang Gung Memorial Hospital, 5, Fu-Shing St., Kwei-Shan, Taoyuan, 333, Taiwan

**Keywords:** Premature infant, Attention-deficit/hyperactivity disorder, Behavioral disorder, DSM-IV, Gestational age

## Abstract

**Background:**

Preterm survivors from the neonatal intensive care unit (NICU) are considered to be at risk for some neurobehavioral disorders such as attention-deficit/hyperactivity disorder (ADHD). The current study aimed to explore the relationship between ADHD and premature infants in Taiwan.

**Methods:**

A total of 195 children (157 males and 38 females) diagnosed with ADHD based on DSM-IV and aged between 6 to 12 years and a control group of 212 (164 males, 48 females) age- and sex-matched healthy children were enrolled. The ADHD-Rating scale and CGI severity were performed by child psychiatrists. Demographic data of the children, including birth history, perinatal neurological and respiratory problems were collected to facilitate the investigation of whether a correlation exists between ADHD and prematurity.

**Results:**

The ADHD group had a significantly higher rate of prematurity and significantly higher rate of low birth body weight (defined as <2500 g) than the control group (both *P* = 0.003). Pearson correlation showed a significantly negative correlation between gestational age and ADHD-RS score, inattentive score, hyperactivity and CGI-S score (P = 0.004, 0.013, 0.015 and 0.002, respectively). However, only a CGI-S score (P = 0.018) showed a significantly correlation between low birth weight and ADHD.

**Conclusions:**

Premature infants have significantly more severe symptoms of ADHD at school age and they were highly correlated. Further study is necessary to determine the main effect and pathogenesis of moderate as well as extreme preterm birth on the development of ADHD.

## Background

Attention-deficit hyperactivity disorder (ADHD), a neurobehavioral syndrome characterized by inattention, impulsivity and hyperactivity, has been described for over a century as an unruly behavioral disorder most commonly seen in boys [1]. ADHD is noted in 3 ~ 9% of children and adolescents with a male-to-female ratio of 3:1 to 5:1 by previous publications [2-4] and 3–5% of adults with an equal male to female ratio based on the American Psychiatric Association’s Diagnostic and Statistical Manual of Mental Disorders IV (DSM-IV) criteria [5-7]. The exact etiologies of ADHD have not been conclusively determined; however, lower brain volume or weight [8] and potential brain damage during perinatal insult of preterm birth [9] were proposed to be the possible reason of poor maturation of brain, which might result in development of ADHD during pre-school age.

There is growing interest in the mental health of premature infants, partly because their mortality rate has decreased over the last two decades [10,11] and most of them survive without major physical disabilities. Very low birth weight infants (VLBW) are reported to be at increased risk of psychosocial [12-15] or behavioral problems [16]. In addition, VLBW children have a higher risk of perinatal white matter injury, which is associated with perceptual, cognitive, motor and mental health impairment [17]. A recent study also suggested that moderately preterm children born at 32 to 36 weeks’ gestational age specifically showed attention difficulties when compared with term-born children at school age [18]. An increase in the rates of preterm birth and low birth weight was found in both the United States and Asia [19,20], and their long-term psychosocial impacts deserve further evaluations. The objective of this study was to analyze the relationship between ADHD and premature infants and the associations between severity of symptoms of ADHD and birth weight or gestational age.

## Methods

### Subjects

We reviewed the medical records of all children diagnosed with ADHD between January 2001 and December 2009 in the Department of Pediatric Psychiatry of Chang Gung Children’s Hospital (CGMH), a tertiary-level university-affiliated teaching hospital in northern Taiwan. This study was approved by the institutional review board of CGMH**.** All patients were diagnosed by expert child psychiatrists and fulfilled the criteria of ADHD according to the 2000 DSM-IV-TR [21]. Children aged between 6 and 12 years old at the time of diagnosis of ADHD were recruited and studied in a regular education framework. We excluded children with an IQ of less than 70 on the WISC-III [22] and those with mental retardation, congenital anomalies, chromosome anomalies and/or neurological disorders.

A group of children involved in the study as the controls were recruited from the general community, and didn’t have any major physical diseases, neurological problems, or psychiatric illnesses. We planned to enroll a group of age and sex-matched children to our ADHD group. An informed consent was obtained from their parents after well explanation. During this study period, 195 ADHD children (157 boys and 38 girls with M/F ratio of 4.1:1; mean age 8.8 years, SD 1.9, range 6–12 years) who were diagnosed based on DSM-IV-TR were enrolled. The control group consisted of 212 age- and sex-matched children (164 boys and 48 girls with M/F ratio of 3.4:1; mean age 9.1 years, SD: 3.9). There was no significant difference between the basic demographic data of the study and control groups (Tables 1).

**Table 1:** Demographic data of ADHD children versus controls

	**ADHD group**	**Control group**	**P value**	**Odds ratio(C.I.)**^**(3)**^	**Odds ratio(C.I.)**^**(4)**^
Case number	195	212			
Age (y/o) (mean ± SD)	8.79 ± 1.93	9.13 ± 3.87	0.488^(1)^		
Gender			0.499^(2)^		
Male, n (%)	157 (80.5)	164 (77.3)			
Female, n (%)	38 (19.5)	48 (22.6)			
Birth body weight, g (mean ± SD)	3007 ± 563.5	3351 ± 417.5	0.011^(1)^	0.999(0.998, 1)	0.999(0.998, 1)
Gestational age, weeks (mean ± SD)	37.6 ± 2.21	38.8 ± 1.37	0.005^(1)^	0.67(0.51, 0.89)	0.67(0.50, 0.89)
Prematurity*, n (%)	27 (18.9)	18 (8.5)	0.003^(2)^	(5)	(5)
Low birth body weight^¶^, n (%)	24 (16.2)	14 (6.6)	0.009^(2)^	(5)	(5)
Respiratory complications at birth, n (%)	5 (2.6)	2 (0.9)	0.336^(2)^		
Neurological complications, n (%)	3 (1.5)	1 (0.5)	0.600^(2)^		
Body mass index (pretest), kg/m^2^	18.1 ± 3.49	18.72 ± 3.67	0.422^(1)^		
ADHD Rating Scale IV					
Total score (mean ± SD)	29.8 ± 7.98	10.73 ± 2.81	< 0.001^(1)^		
Inattention score (mean ± SD)	16.9 ± 4.23	7.1 ± 1.29	< 0.001^(1)^		
Hyperactivity score (mean ± SD)	12.7 ± 5.33	4.17 ± 1.78	< 0.001^(1)^		
ADHD CGI-S (pretest) (mean ± SD)	4.5 ± 0.91	1.15 ± 0.29	< 0.001^(1)^		

*ADHD* attention-deficit/hyperactivity disorder; *SD* standard deviation; *CGI-S* Clinical Global Impression-Severity.

*Prematurity was defined as delivered before 37 weeks’ gestation.

Low birth body weight was defined as < 2500 g.

(1): Independent samples *T* test.

(2): Fisher’s exact test.

(3): Odds ratio and confidence interval in logistic regression with ADHD as response and Birth body weight/Gestational age as the only predictor.

(4): Odds ratio and confidence interval in logistic regression, adjusted for gender and age.

(5): One of the cells in 2 by 2 table is equal to 0, so we only present significance of Fisher’s exact test.

### Procedures

An interview instrument of ADHD Rating Scale IV (ADHD-RS IV) [23], which is a rating scale that provides a reliable means of rating ADHD symptom severity for boys and girls aged 5–17 years old, was used by experienced child psychiatrists. A Clinical Global Impression-Severity (CGI-S) score was used to measure the symptom severity and treatment response of children with ADHD. All birth history of the children in both the ADHD and control groups, including birth weight, gestational age, any perinatal insults, and respiratory or neurological complications were recorded from chart review and analyzed. A child was considered to have respiratory complication if he or she was admitted to the neonatal intensive care unit due to requirement of oxygen or ventilator support at birth.

### Statistical analysis

We defined prematurity as a child who was delivered before 37 weeks’ gestation and low birth body weight as < 2500 g. The chi-square test and Fischer’s exact test were used for categorical data, and comparisons of the continuous data between groups were performed by the student’s *t*-test. Logistic regression was used to calculate odds ratios (ORs) and 95% confidence intervals (CI) for risk of developing ADHD with prematurity and low birth weight. Multivariate logistic regression was performed to adjust for covariates of gender and age. Three kinds of model were tested by simple and multiple regressions. Model 1a, 1b, 1c, 1d analyze the effects of preterm on total score of ADHA, ADHD inattention score, ADHD hyperactivity score and CGI-S scores. Model 2a, 2b, 2c, 2d test the impacts of preterm and LBBW on total score of ADHA, ADHD inattention score, ADHD hyperactivity score and CGI-S scores. For Model 3a, 3b, 3c, 3d, gender and age were added into Model 2a, 2b, 2c, 2d as covariates to control the effects of these two variables. All statistics were performed using the commercially available software SPSS software for Windows (version 13.0; SPSS, Inc., Chicago, IL), and a *P* value of less than 0.05 (two-tailed) was considered to be statistically significant.

## Results

During the study period, a total of 195 children (157 males and 38 females) diagnosed with ADHD based on DSM-IV-TR were enrolled and completed the whole assessments. A control group of 212 age- and sex-matched children (164 males, 48 females) were recruited from the surrounding schools in the community. The demographic data, including birth history, are summarized in Tables 1. The gestational age of the ADHD group and control group were 37.6 ± 2.21 (median: 38, range: 27–43) weeks and 38.8 ± 1.37 (median: 38; range: 36–42) weeks, respectively (P = 0.08). Their mean birth body weight were 3007 ± 563.5 (median: 3015; range: 1700–4350) and 3351 ± 417.5 (median: 3250; range: 2500–4050) g, respectively (P = 0.018). The ADHD group had significantly higher rate of prematurity and higher rate of low birth body weight than the control group (18.5% vs. 8.5%, and 15.8% vs. 6.6%, both *P* = 0.003). The body mass index and rate of respiratory complications or neurological complications at birth were not significantly different between these two groups. The severity of ADHD symptoms, judged by ADHD-RS-IV and CGI-S scores, was significantly more severe in the ADHD group than the control group (all *P*<0.001).

Tables 2 presents The ADHD symptoms severity by Influences of various factors (Preterm birth and low birth body weight). From Tables 2, we could find that when children with preterm birth was only one predictor in regression, it had significant effects on total score of ADHA, ADHD inattention score, ADHD hyperactivity score and CGI-S scores(β = 0.218, p<0.01, β = 0.189, p<0.05, β = 0.167, p<0.05, β = 0.233, p<0.01, respectively)(See model 1a, 1b, 1c, 1d). When adding LBBW into regression, it still had significant effects on total score of ADHA and CGI-S scores, but the effects on ADHD inattention score, ADHD hyperactivity score became not significant(See Model 2a, 2b, 2c, 2d). For LBBW, it had no significant effects on all scores and theβs were far lower that those of children with preterm birth. This result indicated that children with preterm birth had larger influence on the ADHD symptoms severity. When gender and gestational age were also put into regressions as control variables, the results were similar to the second model (Model 2a, 2b, 2c, 2d).

**Table 2:** The ADHD symptoms severity by Influences of various factors (Preterm birth and low birth body weight)

	**Model 1a**	**Model 2a**	**Model 3a**	**Model 1b**	**Model 2b**	**Model 3b**	**Model 1c**	**Model 2c**	**Model 3c**	**Model 1d**	**Model 2d**	**Model 3d**
	**ADHD Total score**			**Inattention score**			**Hyperactivity score**			**ADHD CGI-S**		
Preterm	0.218**	0.218*	0.194*	0.189*	0.167	0.153	0.167*	0.178	0.148	0.233**	0.201*	0.181*
LBBW		−0.003	0.002		0.037	0.041		−0.023	−0.015		0.051	0.057
Gender			−0.131			−0.078			−0.165*			−0.107
age			−0.137			−0.077			−0.162*			−0.131
F-value	7.943**	3.847*	3.506**	5.899*	2.924	1.95	4.570*	2.234	3.434*	9.436**	4.73*	3.676**
Adj.R^^^2	0.042	0.047	0.059	0.030	0.024	0.02	0.022	0.015	0.058	0.048	0.043	0.061

*: p-value <0.05; **: p-value <0.01.

*ADHD* attention-deficit/hyperactivity disorder.

*CGI-S* Clinical Global Impression-Severity.

Preterm: indicator variable (“Gestational age < 37 weeks” = 1; otherwise = 0).

LBBW: indicator variable (“Birth body weight < 2500 g” = 1; otherwise = 0).

Gender: control variable (“male” = 0; “female” = 1).

Age: control variable.

F-value: F statistics of ANOVA.

*Adj.R*^*^*^*2* adjusted R-squared.

Tables 3 showed the Pearson correlation performed for evaluation if ADHD symptoms severity is associated with preterm birth or birth body weight. Gestational age was noted to be negatively correlated with severity of ADHD symptoms, and all parameters, including ADHD RS total score, inattention or hyperactivity scores and ADHD CGI-S scores, were found to reach a significant level of less than 0.05. Birth body weight was noted to be negatively correlated with ADHD CGI-S score (Pearson correlation, r =−0.181, *P* = 0.018), but was not significantly correlated with hyperactivity score. Moreover, a lower birth body weight was also found to correlate with a higher ADHD RS total (P = 0.057) and inattention (P = 0.053).

**Table 3:** The associations of ADHD symptoms severity and birth body weight and gestational age evaluated by Pearson correlations

**Pearson correlation**	**Gestational age (weeks)**	**Birth body weight (g)**
ADHD RS total score		
r	−0.225	−0.149
p	0.004**	0.057
ADHD inattention score		
r	−0.195	−0.152
p	0.013*	0.053
ADHD hyperactivity score		
r	−0.192	−0.115
p	0.015*	0.143
ADHD CGI-S (pretest)		
r	−0.237	−0.181
p	0.002**	0.018**

*ADHD* attention-deficit/hyperactivity disorder.

*CGI-S* Clinical Global Impression-Severity.

## Discussion

Our study found that the ADHD group has a relatively higher rate of prematurity and a significantly higher rate of low birth body weight than the general population. We also demonstrated that both moderate preterm birth (gestational age between 33 weeks to 37 weeks) and low birth body weight (defined as birth body weight < 2500 g) are associated with more severe ADHD symptoms. Previous studies have concluded that very preterm children were reported to exhibit significantly more behavioral problems and cognitive disorders [16,24]; however, most of these studies focused on very preterm (≤ 33 weeks’ gestation) and extremely very low birth body weight [24-27]. Our data support recent literature with regard to the observation that inattention and emotional regulation difficulties affect the functioning of moderate preterm children at school age [18].

The significant association between ADHD and preterm birth or low birth body weight can be explained by several mechanisms. First, some of the children suffered from intrauterine growth retardation and their less matured brain structure due to suboptimal fetal environment; these conditions may be associated with postnatal illness and later development of ADHD [28]. Second, less favorable parent–child or family interaction with children during the first half-year of preterm or SGA (small for gestational age) [29] may affect the neurobehavioral development of these children. Furthermore, the underlying causes of premature birth, either genetic or environment factors, may also influence or interfere with normal neuronal development and organizations, which may contribute to subsequent ADHD symptoms [30].

Another interesting finding of our study was that the more severe ADHD symptoms, including inattention, hyperactivity and impulsivity, were significantly correlated with more preterm birth or lower gestational age. However, lower birth body weight was only associated with inattention, but not hyperactivity or impulsivity. To our knowledge, our study was the only one to conclude both low birth body weight and preterm birth as independent risk factors for the development of ADHD at school age. These two factors may not be correlated, because in studies of low-birth-weight (<2500 g) but term-born children, small body size at birth predicts behavioral symptoms of ADHD [28,31]. These findings can be partially explained by the theory that inattention was correlated with cognition and hyperactivity or impulsivity was correlated with poor inhibition of movement. Low gestational age could affect both neurological cognition and control of movement, while birth body weight might only affect cognition. This theory deserves further prospective design with larger sample to confirm this theory.

Previous studies have demonstrated an increased risk for ADHD in follow-up studies of preterm survivors from NICUs [32-34]. Even in moderately preterm children, cognitive and emotional regulation difficulties affect their functioning at school age, and a slightly lower IQ with attention and behavioral problems are found when they are compared with term-born children [35,36]. In these studies, inattention problems are found in 15–25% of the moderately preterm-born children and approximately one-third of very preterm children at their school age [32-36]. In addition, preterm birth carries some risk for psychiatric disorders requiring hospitalization in adolescence and young adults [33,37], thus the requirement of more attention in research and secondary prevention is warranted.

A major limitation of our study is its relatively small sample size and retrospective design. We are unable to further extend our conclusion to very low birth body weight children (< or = 1500 g) and very preterm children (< or = 32 weeks’ gestation). However, our study had excluded the cases of well-defined brain damage such as intraventricular hemorrhage, periventricular leukomalacia, or cerebral palsy from perinatal asphyxia. We also excluded children with an IQ of less than 70 on the WISC-III, and those with major mental, neurological, or physical disease, which may have resulted in the exclusion of very low birth body weight and very preterm infants from our study sample. Furthermore, our study applied ADHD questionnaires from parents and teachers rather than utilizing objective measurements of quality of life, person-to-person relationships, academic achievements, or neurocognitive tests. A further well-designed, prospective study is required to confirm our findings.

It is our hope that, by gaining a better understanding of the strong relationships between preterm birth, low birth body weight and the risk of developing ADHD, many undefined efforts can be progressed to avoid preterm birth. Further attention should be paid to these children by having child psychiatric clinics perform regular follow-ups to track their behavioral and emotional conditions.

## Conclusion

Children with ADHD have a significantly higher rate of prematurity and low birth body weight. The severity of ADHD symptoms, including ADHD RS total score, inattention or hyperactivity scores and ADHD CGI-S scores, are highly correlated with extent of prematurity. Premature brain, less favorable postnatal condition and illness of preterm neonates, or poor parent-child interaction may contribute interactively to their suboptimal neurobehavioral development and subsequent ADHD, but the underlying pathogenesis remains uninvestigated and deserves further studies.

## Competing interests

The authors declare that they have no competing interests.

## Authors’ contributions

S-MC Carried out the study and wrote the manuscript. M-HT collected the data; assumed the role of attending physician of the premature neonates. J-FH Responsible for editing the English version of the study; assumed the role of attending physician of the premature neonates. F-MH performed the statistical analysis. H-RH the attending physician of our premature neonates. Y-SH the corresponding author, overview and supervise the whole study and writing of manuscript. All authors read and approved the final manuscript.

## Pre-publication history

The pre-publication history for this paper can be accessed here:

http://www.biomedcentral.com/1471-244X/12/85/prepub
